# Tissue shape from cell-scale active tensions

**Published:** 2026-10-15

**Authors:** Nikolas H. Claussen, Fridtjof Brauns, Boris I. Shraiman

## Abstract

Connecting cell and tissue mechanics is a fundamental challenge in the physics of morphogenesis. Conventional elasticity theory is generally inapplicable: cytoskeletal turnover precludes a fixed reference state, and the dominant forces are generated actively, independently of strain. Here, we study epithelia in quasi-static force balance between intracellular pressure and active interfacial tensions. The theory starts from a prescribed set of active tensions, represented as a Maxwell-Cremona tension triangulation dual to the cell tiling. This geometric formulation brings to bear the mathematics of discrete conformal geometry. Using generalized Voronoi diagrams, we construct the mechanically balanced cell tiling resulting from the given tensions and compute the tissue-scale stress. Conceptually, we treat cell interfaces as force dipoles whose embedding in physical space -the cell tiling- is determined by force balance. The tensions define an effective reference state in which the macroscopic stress vanishes as pressures and tensions cancel locally. Deformations from this reference result in macroscopic stress, constituting an emergent form of elasticity. Adiabatic tension dynamics change the reference shape. Thus, rather than fluidizing, tissues can deform -or "morph"-, while resisting external forces like a solid. We also define a conformal-geometric representation of cell network topology, remodeled by plastic cell rearrangement. We make experimentally testable predictions, notably that macroscopic stress is independent of local tension anisotropy. Overall, the unified description of emergent elasticity and plastic flow in epithelia is a step towards a theory of morphogenesis that puts active forces first and reflects the differences between inert and biological matter. Our framework also applies to 2D fluid foams and to frictionless granular media via a mechanical duality.

